# Turmeric (*curcuma longa*) rhizome essential oil: analytical profile of authenticated and commercial samples, safety and pharmacology review

**DOI:** 10.1080/13880209.2026.2629622

**Published:** 2026-03-01

**Authors:** Diana I. Saavedra, Dakota T. Carter, Joseph T. Dawson, Sara A. Shah, Nicole Stevens, Ambika Poudel, Prabodh Satyal, Cécile Bascoul

**Affiliations:** dōTERRA International, Pleasant Grove, UT, USA

**Keywords:** *Curcuma longa*, turmeric, essential oil, safety, pharmacology, GC-MS

## Abstract

**Context:**

Turmeric (*Curcuma longa*) rhizome essential oil is the volatile and aromatic fraction of turmeric rhizome. Despite increasing commercial availability and scientific interest, variability in production practices and oil composition has created challenges for quality, safety, and pharmacological interpretation.

**Objective:**

This work provides a comprehensive overview of turmeric rhizome essential oil including an assessment of the composition of authentic oils, and commercial oils, and a review of safety and pharmacology.

**Materials and methods:**

Authentic samples from India (*n* = 35) and Nepal (*n* = 15), and market samples (*n* = 25) were analyzed by GC-MS. A review of published toxicological and pharmacological studies was conducted.

**Results:**

Authenticated oils were primarily composed of turmerones. Sesquiterpenes were more abundant in the oil from ground material, whereas monoterpenes were more prevalent in the oil from fresh rhizomes. Most market samples also exhibited the large dominance of turmerones, with some showing compositional variability, dilution, or adulteration, including 4/25 samples containing adulteration markers. Toxicological studies indicate no genotoxicity or dermal toxicity, minimal systemic toxicity, and good tolerability in humans. The pharmacology data supports anti-inflammatory, antimicrobial, neuroprotective, antioxidant, and anticancer properties, with synergistic effects when turmerones and curcuminoids are combined.

**Discussion and conclusion:**

Turmeric rhizome essential oil demonstrated a favorable safety profile and diverse bioactivities with therapeutic potential. However, compositional variation in commercial products with adulteration highlights the need for production standards. Authentic and consistent essential oil composition is key to ensure reproducible research findings, validate therapeutic potential, guarantee safety and provide the desirable outcomes when used.

## Introduction

### Botanical source and historical use

Turmeric (*Curcuma longa* L.; synonym: *Curcuma domestica* Valeton) is a rhizomatous herbaceous perennial flowering plant which belongs to the Zingiberaceae family and *Curcuma* genus (Prasath et al. 2018). The plant is native to tropical South Asia and has been widely introduced and naturalized to many tropical and subtropical countries. India produces most of the world’s supply, but turmeric is also cultivated in other countries in South, East and Southeast Asia as well as in the Americas (Plotto et al. 2004).

The use of turmeric dates back nearly 4000 years to the Vedic culture in India, where it was used as a culinary spice and had some religious significance. It reached China by 700 AD, East Africa by 800 AD, West Africa by 1200 AD, and Jamaica in the eighteenth century. Turmeric has been used in folk medicine preparations over the centuries in different parts of the world. In Ayurvedic practices, turmeric is thought to have many medicinal properties, including strengthening the overall energy of the body, relieving gas, dispelling worms, improving digestion, regulating menstruation, dissolving gallstones, and relieving arthritis (Prasad and Aggarwal 2011). Turmeric rhizomes contain two major classes of secondary metabolites: curcuminoids, which are responsible for the yellow color of the turmeric, and turmerones, which contribute to the characteristic aroma and flavor (Parthasarathy et al. 2008). While turmeric has been extensively studied along with curcumin, as one of the main active constituents (Sharifi-Rad et al. 2020), the interest and use of the essential oil of turmeric have been growing.

Turmeric rhizomes contain about 0.5-6.0% essential oil (Guenther 1992; WHO 1999; Parthasarathy et al. 2008; Thaikert and Paisooksantivatana 2009; EFSA FEEDAP 2020). The oil is extracted through steam distillation or hydrodistillation. Sesquiterpenes, including ar-turmerone, α-turmerone, and β-turmerone, are the major constituents present in the oil (Guenther 1992; Dosoky et al. 2019; Jaiswal and Naik 2021). Other notable compounds include ar-curcumene, β-sesquiphellandrene, α-zingiberene, α-atlantone, p-cymene, 1,8-cineole, γ-curcumene. The oil of turmeric is traditionally used as an antacid, and in small doses, acts as a carminative, stomachic, appetizer, and tonic (CSIR 1950). It is used as a flavoring for food products and in animal feed. It has also been used in perfumes of heavy oriental character (Guenther 1992). Turmeric essential oil is widely utilized in cosmetic and pharmaceutical applications due to its antimicrobial, anti-inflammatory, antioxidant, and insect-repelling properties (Orellana-Paucar 2024). Moreover, some components of turmeric oil can be converted into compounds of perfumery value through appropriate chemical reactions (Banerjee et al. 1981).

### Sourcing and production methods

Turmeric essential oil is produced from cultivated rhizomes. Growing conditions, such as water availability, sun exposure, and harvest time affect rhizome quality which can have subsequent effects on the quality of the essential oil. Yan et al. reported a 16% increase in the relative abundance of ar-turmerone in essential oils from shade grown turmeric compared to turmeric grown in full sun, despite no significant difference in extraction yield (Yan et al. 2016). Cooray et al. found that the volatile oil content per 100 g of dried rhizome is greatest at 5 months after planting; although, the ideal harvest time for essential oil production is between 7.5 and 8.0 months after planting when the total yield per turmeric bush is the greatest (Cooray et al. 1988). Additionally, installation of deep irrigation systems improved turmeric crop quality in the Rautahat municipality of Nepal compared to the dryland farming techniques previously utilized, resulting in improvements in essential oil quality (Adhikari 2025).

Turmeric essential oil is distilled from fresh, dried, or cured rhizomes (Kutti Gounder and Lingamallu 2012; Ray et al. 2022). After harvesting, the rhizomes are thoroughly cleaned with water to remove soil and debris. Fresh rhizomes are then cut into smaller pieces or pureed depending on the extraction process. Dried rhizomes are typically dehydrated whole and pulverized before essential oil extraction. This material is also known as and referred to in subsequent sections of this paper as essential oil from “ground turmeric rhizome.” For curing, fresh rhizomes are boiled in water (∼1 h), strained, and dried in the shade (∼1 week).

The overall yield and relative abundance of monoterpenes is significantly greater in essential oils extracted from fresh rhizomes compared to dried rhizomes (Kutti Gounder and Lingamallu 2012; Ray et al. 2022). Ray et al. reported that this does not apply to essential oils derived from freeze dried rhizomes, which were found to be compositionally similar and to have a greater yield than fresh rhizome essential oils (Ray et al. 2022). Higher levels of ar-turmerone and lower levels of α-turmerone were found in essential oils extracted from rhizomes that underwent hot air oven drying processes compared to essential oils from fresh rhizomes (Kutti Gounder and Lingamallu 2012; Ray et al. 2022). Ray et al. postulate that this is caused by the conversion of α-turmerone to the more energetically favorable ar-turmerone from the application of heat during the drying process. However, this theory does not fully explain why the ar-turmerone concentration is 2x greater than the α-turmerone concentration in essential oils from shade dried rhizomes, nor why essential oils from solar dried rhizomes have ar-turmerone and α-turmerone levels that resemble essential oils derived from fresh rhizomes (Ray et al. 2022).

Turmeric essential oil is extracted from rhizomes *via* hydrodistillation and steam distillation methods. During hydrodistillation, pulverized dried rhizomes or pureed fresh rhizomes are mixed with water and boiled in the round bottom flask of a Clevenger apparatus or in the boiler of a manufacturing scale hydrodistillation system. The essential oil and water are collected in a condenser and subsequently separated (Ibáñez and Blázquez 2020). In contrast, during steam distillation, the plant material is placed on a perforated plate and blasted with steam thereby evaporating the material’s steam-volatile compounds. Then the evaporated water and volatile oil are collected in a condenser and are separated mechanically (Akdağ and Öztürk 2019; Ibáñez and Blázquez 2020). Following separation, both hydrodistilled and steam distilled oils are filtered and treated with anhydrous sodium sulfate, which acts as a drying agent to remove any remaining water in the essential oil (Kutti Gounder and Lingamallu 2012; Yan et al. 2016; Pham and Le 2020; Ray et al. 2022). Hydrodistillation is by far the most common method for extracting turmeric essential oil on both laboratory and industrial scales, as it is more economical than steam distillation, which requires larger amounts of raw material and longer extraction times (Ibáñez and Blázquez 2020; Masango 2005). Tiwari et al. report that hydrodistilled and steam distilled turmeric essential oils differ in color value but are relatively similar in terms of specific gravity, refractive index, ester value, acid value, and saponification value (Tiwari et al. 2022). Essential oil compositions reported in the literature are far more diverse for steam distilled essential oils than hydrodistilled essential oils, but it is unclear if this is due to variation in the method of analysis or other differences in study approaches between researchers (Chane-Ming et al. 2002; Braga et al. 2003; Chang et al. 2006; Asghari et al. 2009; Kutti Gounder and Lingamallu 2012; Yan et al. 2016; Mustafa Maarof 2018; Pino et al. 2018; Pham and Le 2020; Mc Gaw and Skeene 2021; Poudel et al. 2022; Ray et al. 2022; Tiwari et al. 2022; de Souza Junior et al. 2023).

Standardization in essential oil production is achieved by controlling harvest timing, rhizome maturity, and distillation parameters. Process optimization focuses on maximizing yield while maintaining a consistent chemical profile of key constituents (ar-turmerone, α-turmerone, β-turmerone). Quality control includes monitoring moisture content, performing thin-layer chromatography (TLC), assessing acid value, and conducting gas chromatography-mass spectrometry (GC-MS) to confirm the presence of active compounds (Li et al. 2011).

This work aims to provide a comprehensive overview of turmeric (*C. longa*) rhizome essential oil through an assessment of the composition of authentic oils from fresh and ground turmeric rhizomes in relation to sourcing and production practices, an evaluation of the authenticity of oils available on the market, and a review of the current knowledge regarding safety and pharmacological properties, thereby highlighting the impact of sourcing and production practices on research and uses of the oil.

## Materials and methods

### Turmeric essential oil sample selection

To establish the typical chemical composition of authenticated turmeric (*C. longa*) rhizome essential oils, reference samples with documented plant part, extraction method, country of origin, and manufacturer were characterized. Authenticated samples of fresh turmeric essential oil from Nepal (*n* = 15) and ground turmeric essential oil from India (*n* = 35) were collected from raw material suppliers and evaluated.

Market samples were identified on Amazon.com using the search term “turmeric oil.” All products claiming to be 100% turmeric oil were acquired. Labels were evaluated upon receipt of the product to assure that turmeric oil was the only ingredient listed. All label information was recorded including brand, ingredients, instructions, cautions, product claims, and an image of the product. Altogether 25 market samples were found to meet the study criteria. These were coded and sent for GC-MS analysis.

Table 1 summarizes all turmeric samples analyzed, both authenticated samples and commercial samples.

**Table 1.** Turmeric essential oil samples.

Sample Type	Sample Size	Source	Country of Origin
Turmeric Fresh Rhizome Essential Oil	n = 15	dōTERRA	Nepal
Turmeric Ground Rhizome Essential Oil	n = 35	dōTERRA	India
Market Survey	n = 25	Amazon.com	Various

### GC-MS analysis

Essential oils were analyzed by GC-MS using a Shimadzu GCMS-QP2010 Ultra operated in the electron ionization (EI) mode (electron energy = 70 eV), scan range = 40–400 atomic mass units, scan rate = 3.0 scans/s, and GC-MS solution software (Shimadzu Scientific Instruments, Columbia, MD, USA). The GC column was a ZB-5 fused silica capillary column with a (5% phenyl)-polymethylsiloxane stationary phase and a film thickness of 0.25 μm, a length of 30 m, and an internal diameter of 0.25 mm (Phenomenex, Torrance, CA, USA). The carrier gas was helium with a column head pressure of 552 kPa and flow rate of 1.37 mL/min. The injector temperature was 250 °C and the ion source temperature was 200 °C. The GC oven temperature was programmed for 50 °C initial temperature, then temperature was increased at a rate of 2 °C/min to 260 °C. A 7% w/v solution of the sample was prepared in dichloromethane and 0.1 μL was injected with a splitting mode (30:1). Identification of the oil components was based on their retention indices determined by reference to a homologous series of n-alkanes, and by comparison of their mass spectral fragmentation patterns with those reported in the literature (Adams 2007) and an in-house library (Satyal 2015).

### Literature review of turmeric essential oil safety and pharmacology

Structured literature searches were conducted between June 2025 and November 2025 using Google Scholar and PubMed to identify studies evaluating turmeric essential oil and its major constituents, particularly turmerones. Initial searches using the terms ‘turmeric essential oil’ and ‘turmerones’ yielded approximately 5,160 records. ‘turmeric oil’, ‘*Curcuma longa* oil’ and ‘*Curcuma longa* essential oil’ were also used. All articles found is the search were evaluated. Abstracts were screened for relevance, and studies primarily focused on curcuminoids or nonvolatile turmeric extracts and preparations were excluded. For the safety review, key endpoints examined in the review encompassed dermal toxicity such as skin irritation, sensitization, and phototoxicity, as well as acute and chronic toxicity, genotoxicity, and reproductive and developmental toxicity. Additionally, relevant clinical trials and case reports were considered to support the overall safety evaluation. For the pharmacology review, targeted searches were subsequently performed by combining “turmeric essential oil” and “turmerones” with pharmacological activity-related terms, including antimicrobial, anti-inflammatory, cognitive support, cancer, and bioavailability. Reference lists of the most relevant articles and review papers were manually examined to identify additional primary sources, and cited studies were traced back to their original publications where applicable. Full-text articles were reviewed, and those most relevant to the scope of turmeric essential oil and turmerone were selected for inclusion. Limitations of this review include the potential omission of relevant studies, exclusion of non-English-language publications, and under-representation of emerging research that may not yet be fully indexed in major databases.

## Results and discussion

Representative chromatograms of ground and fresh turmeric rhizome essential oil and adulterated samples MAR008, MAR017, MAR013, and MAR019 are available in Supplementary Figure S1.

### Turmeric essential oil typical composition

Compositional data in the tables below report the median values of 15 GC-MS reports on fresh turmeric rhizome essential oil, sourced from Nepal (Table 2), and of 35 GC-MS reports on ground turmeric rhizome essential oil, sourced from India (Table 3).

**Table 2.** Main constituents of authenticated fresh turmeric (*C. longa*) rhizome essential oil, sourced from Nepal (%, data representing the median values of the top 25 constituents identified *via* GC-MS).

Compound	Med. (%)
α-Turmerone	28.46
ar-Turmerone	19.63
β-Turmerone	13.45
Terpinolene	7.74
α-Zingiberene	4.85
β-Sesquiphellandrene	4.72
Helifolen-12-al b	2.85
trans-β-Caryophyllene	2.27
2,4,4,6-Tetramethyl-6-phenyl-1-heptene	2.01
trans-α-Atlantone	1.63
ar-Curcumene	1.49
Biotol	1.17
6R,7R-Bisabolone	0.86
β-Bisabolene	0.72
1,8-Cineole	0.55
α-Humulene	0.48
α-Terpinene	0.45
Sesquisabinene hydrate	0.43
trans-Nerolidol	0.43
ar-Turmerol	0.39
α-Phellandrene	0.37
Curcuphenol	0.35
cis-α-Atlantone	0.35
Zingiberenol	0.34
Megastigma-4,6,8-triene	0.28

**Table 3.** Main constituents of authenticated ground turmeric (*C. longa*) rhizome essential oil, sourced from India (%, data representing the median values of the top 25 constituents identified *via* GC-MS).

Compound	Med. (%)
ar-Turmerone	28.98
α-Turmerone	23.07
β-Turmerone	15.50
α-Zingiberene	4.68
β-Sesquiphellandrene	4.03
ar-Curcumene	3.41
Helifolen-12-al b	2.41
trans-α-Atlantone	1.80
Isobicyclogermacrene	1.76
2,4,4,6-Tetramethyl-6-phenyl-1-heptene	1.68
Biotol	1.17
β-Bisabolene	1.04
trans-β-Caryophyllene	0.70
trans-γ-Atlantone	0.63
Germacrene B	0.55
cis-γ-Atlantone	0.52
cis-α-Atlantone	0.46
6R,7R-Bisabolone	0.45
α-Santalene	0.39
γ-Curcumene	0.39
Curcuphenol	0.36
ar-Turmerol	0.36
β-Curcumene	0.24
Curcumadione	0.22
1,8-Cineole	0.20

In the authenticated samples tested, the twenty-five top constituents represented approximately 95% of the essential oils. Both the fresh and ground rhizome essential oils contained large amounts (>85%) of sesquiterpenoids, including ar-turmerone, α-turmerone, and β-turmerone. Although the oils were produced from two different origins (Nepal and India), and both processing methods and growing conditions influence the final product, it is still informative to compare the essential oil compositions of fresh and ground turmeric rhizomes. Fresh turmeric oil was found to have a larger monoterpene fraction than ground turmeric oil samples, with constituents such as terpinolene (7.74%), 1,8-cineole (0.55%), α-terpinene (0.45%), and α-phellandrene (0.37%). Ground turmeric essential oil was found to have larger amounts of sesquiterpenes such as ar-curcumene (3.41%), isobicyclogermacrene (1.76%), and germacrene B (0.55%). These compositional shifts are consistent with previous findings (Kutti Gounder and Lingamallu 2012; Ray et al. 2022) and reflect the highly volatile and chemically labile nature of monoterpenes, which are prone to significant loss during post-harvest handling. Drying, grinding, and subsequent storage conditions likely facilitated evaporation and oxidative degradation of monoterpenes such as terpinolene, 1,8-cineole, and terpinene, which were more abundant in the fresh rhizome samples. Additionally, the mechanical disruption associated with grinding increases surface area and enhances exposure to oxygen and light, further accelerating monoterpene depletion. In contrast, sesquiterpenes, generally being less volatile, are comparatively well-preserved during dehydration and milling. As a result, constituents such as ar-curcumene, and germacrene B appeared in higher relative concentrations in the essential oils derived from ground turmeric.

### Turmeric essential oil of commercial samples

**Figure 1.**
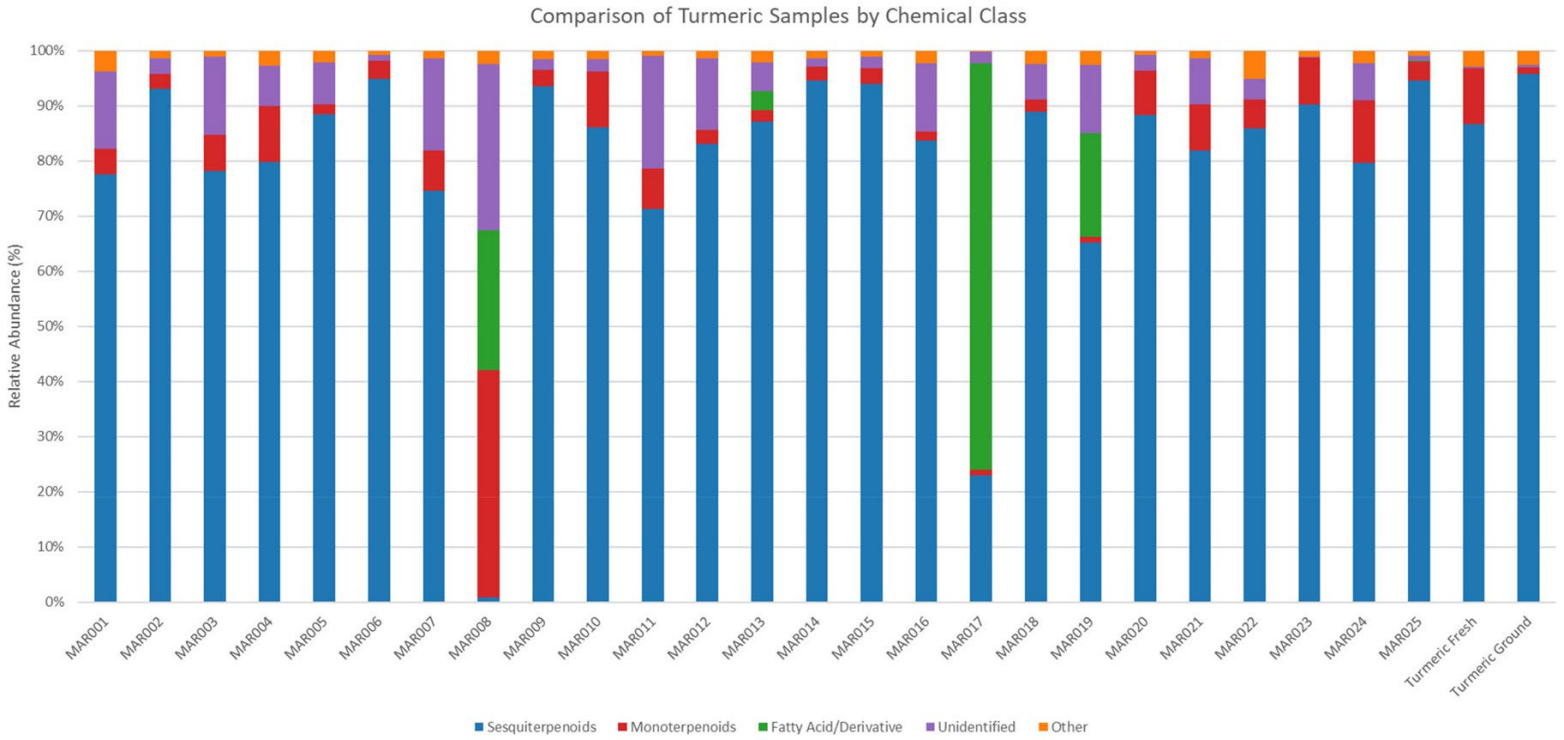
A graphical representation of the relative abundance of chemical classes found in the 25 commercial market samples, and the authenticated turmeric (*C. longa*) rhizome essential oils.

A total of 25 commercial samples purchased online in March 2025 of turmeric (*C. longa*) essential oil were analyzed by GC-MS to assess compositional makeup and market trends. All market samples were advertised and labeled as 100% turmeric essential oil. GC-MS results, including constituents present at ≥0.5% in at least one sample, and unique compounds, of the twenty-five commercial market samples, are available in Supplementary Table S1. Figure 1 represents the relative abundance of chemical classes found in the commercial market samples, and the authenticated turmeric rhizome essential oils. Table 4 is a summary overview of the GC-MS results.

**Table 4.** An overview of the GC-MS results of the commercial market samples including the relative abundance of the three major turmerones, ar-turmerone, α-turmerone β-turmerone, the unidentified fraction, and constituent observations.

Sample	ar-Turmerone (%)	α-Turmerone (%)	β-Turmerone (%)	Unidentified fraction (%)	Constituent Observations
Turmeric Fresh	19.63	25.82	13.45	0.32	Typical turmeric volatiles; high terpinolene
Turmeric Ground	28.92	16.62	15.64	0.42	Balanced sesquiterpene profile
MAR001	23.18	14.08	5.91	13.97	Low sesquiterpene profile; various unidentified constituents
MAR002	28.35	18.28	17.44	2.77	Typical sesquiterpene profile
MAR003	24.33	13.75	6.03	14.15	Low sesquiterpene profile; various unidentified constituents
MAR004	21.66	13.13	6.40	7.34	Low sesquiterpene profile; unidentified components; elevated p-cymene and α-phellandrene
MAR005	28.89	18.25	12.85	7.60	Sesquiterpenes typical of turmeric; some unidentified constituents
MAR006	25.07	14.50	12.59	1.01	Typical turmeric sesquiterpene and overall profile
MAR007	23.28	9.99	4.15	16.69	Low sesquiterpene profile; large number of unidentified constituents
MAR008	0.18	0.12	0.30	30.24	Citral-type components; monoterpene enrichment; fatty acids/esters present; numerous unidentified constituents
MAR009	29.37	14.66	15.16	1.96	Sesquiterpene and overall profile within expected natural variation
MAR010	23.65	12.88	12.18	2.22	Elevated 1,8-cineole and monoterpenes
MAR011	20.90	9.95	4.07	20.36	Low sesquiterpene profile; numerous unidentified constituents
MAR012	25.38	20.06	13.41	12.94	Sesquiterpene profile within expected natural variation; some unidentified compounds
MAR013	21.44	23.68	13.05	5.15	Sesquiterpene profile within expected natural variation; fatty acids/esters present
MAR014	33.37	15.25	14.53	1.50	Typical turmeric sesquiterpene profile
MAR015	33.62	17.15	16.26	2.10	Typical turmeric sesquiterpene profile
MAR016	25.68	23.78	8.82	12.28	Sesquiterpene profile within expected natural variation; some unidentified compounds
MAR017	4.34	8.05	4.10	2.03	Octan-2-yl palmitate (>70%); fatty ester; low monoterpenes
MAR018	32.17	22.14	14.59	6.36	Typical turmeric sesquiterpene profile, some unidentified constituents
MAR019	18.37	18.36	9.75	12.33	Unidentified constituents; minor triglycerides; low monoterpenes
MAR020	27.63	13.37	12.04	2.88	1,8-Cineole elevated; elevated monoterpene fraction
MAR021	32.05	9.55	4.08	8.41	Sesquiterpenes variations; unidentified constituents; elevated monoterpene fraction
MAR022	29.42	12.30	5.94	12.84	Sesquiterpenes variations; unidentified constituents
MAR023	26.55	13.51	12.77	0.18	1,8-Cineole and monoterpenes slightly elevated
MAR024	17.36	15.55	7.55	6.72	Low sesquiterpene profile; minor 1,8-cineole; elevated p-cymene and α-phellandrene; some unidentified constituents
MAR025	17.85	33.78	15.80	0.85	Sesquiterpene profile within expected natural variation

Many samples demonstrated sesquiterpene profiles consistent with authenticated turmeric rhizome essential oil chemistry. The expected dominance of ar-turmerone, α-turmerone, and β-turmerone, along with typically observed minor sesquiterpenes such as α-zingiberene, β-sesquiphellandrene, and ar-curcumene was observed in twenty-two of the commercial samples (Table 5). Examples include MAR002, MAR006, MAR009, MAR014, MAR015 and MAR025. These oils presented the characteristic *C. longa* sesquiterpene signature and had relatively low unidentified fractions, suggesting that they are either genuine turmeric oils or close to authentic compositions.

**Table 5.** Relative abundance ranges of the ten predominant constituents cross twenty-two commercial market samples of turmeric essential oil.

Compound	Range (%)
ar-Turmerone	17.36-33.62
α-Turmerone	9.55-33.78
β-Turmerone	4.07-17.44
α-Zingiberene	3.84-10.63
β-Sesquiphellandrene	2.43-8.77
ar-Curcumene	2.84-8.77
trans-α-Atlantone	1.13-3.65
β-Bisabolene	0.81-2.57
1,8-Cineole	0.45-3.71
γ-Curcumene	0.08-4.45

A subset of samples contained components such as fatty acids, fatty esters, di- and monoacylglycerols, and monoterpenoids atypical to turmeric rhizome essential oil. This occurrence is possibly indicative of dilution or blending with other materials, such as coconut, palm or vegetable oil, other essential oils, or cosmetic-type diluents (Do et al. 2015). Sample MAR017 showed exceptionally strong evidence of deliberate dilution or extension (Table 6). Although turmerones were present (ar-turmerone 4.34%, α-turmerone 8.05%, β-turmerone 4.10%), the chromatogram was dominated by octan-2-yl palmitate at 73.50%, a long-chain ester absent from authentic turmeric oil and commonly used in fragrance or cosmetic formulations. This composition is incompatible with that of genuine *C. longa* essential oil, indicating that the product is not a true essential oil but rather turmeric essential oil diluted in a nonvolatile ester carrier.

**Table 6.** The top 20 compounds found in the market sample MAR017, which includes a large proportion of fatty acid esters, not present in authenticated turmeric rhizome essential oils.

Market Sample MAR017	
Compound	%
Octan-2-yl palmitate	73.50
α-Turmerone	8.05
ar-Turmerone	4.34
β-Turmerone	4.10
Unidentified	2.03
β-Sesquiphellandrene	1.75
α-Zingiberene	1.68
ar-Curcumene	0.71
trans-α-Atlantone	0.62
β-Bisabolene	0.30
1,8-Cineole	0.28
6S,7R-Bisabolone	0.27
Terpinolene	0.25
α-Phellandrene	0.21
β-Caryophyllene	0.18
2,4,4,6-Tetramethyl-6-phenyl-1-heptene	0.15
Biotol	0.14
γ-Curcumene	0.10
1-Hydroxy-3-(octanoyloxy)propan-2-yl decanoate	0.10
Isopropyl palmitate	0.10

Several oils contained very large unresolved fractions, notably MAR008 (30.24% unidentified) (Table 7), MAR011 (20.36% unidentified) and MAR007 (16.69% unidentified). More research is needed to clarify the composition of these unidentified fractions. Although these samples contained some amounts of turmerones, the substantial unidentified portion and irregular monoterpene patterns suggest the presence of other essential oils, synthetic constituents, or degraded components. These profiles are inconsistent with the chromatograms of authentic turmeric oils.

**Table 7.** The top 20 compounds found in the market sample MAR008, which contains atypical monoterpenoids, monoterpenoid esters, and fatty acids not present in authenticated turmeric rhizome essential oils.

Market Sample MAR008	
Compound	%
Unidentified	30.24
Geranial	14.51
Geranyl oleic acid	7.65
Neral	7.56
Citronellal	4.64
Oleic Acid	4.38
Limonene	4.19
Linoleic acid	3.93
Citronellyl acetate	3.54
Neryl palmitic acid	2.15
Citronellic acid	1.75
Citronellyl palmitoleic acid	1.33
Geranyl acetate	1.06
2-trans-4-trans-Decadienal	0.97
trans-Squalene	0.94
Isopropyl myristate	0.92
6-Methyl-5-hepten-2-one	0.91
Geranyl stearic acid	0.81
Geranyl Linolenic acid	0.77
Linalool	0.66

A different pattern was observed in samples enriched in monoterpenes, particularly oxygenated monoterpenes associated with citral- and cineole-rich oils. MAR008 for example, contained citral-type aldehydes (geranial and neral) at unusually high levels for turmeric oil (e.g., MAR008: geranial 14.51%; neral 7.56%), together with citronellal and other related constituents (Table 7). This profile might be indicative of substitution or blending with lemongrass/citronella-type oils rather than pure *C. longa* essential oil. Citral was also reported in turmeric essential oil as the red type (Chowdhury et al. 1970). In addition, several samples (e.g., MAR010, MAR020, MAR023) displayed elevated 1,8-cineole (up to 3.71%) and monoterpene hydrocarbons, alongside reduced or imbalanced turmerone patterns. Although such levels or higher of 1,8-cineole have been reported in turmeric essential oil from rhizome (Dosoky and Setzer 2018), it is mainly present in the essential oil from the leaf and the flower. α-Phellandrene, terpinolene and 1,8-cineole are usually most abundant in the oil from the leaf (Raina et al. 2005; Babu et al. 2007; Awasthi and Dixit 2009; Akbar et al. 2015) while a study reported the concentrations of key constituents in the flower oil including 26% p-cymene, 7.6% terpinolene, and 4.1% 1,8-cineole (Leela et al. 2002).

Finally, several commercial oils, including MAR019 (Table 8), MAR013, and the previously mentioned MAR008 and MAR017, contained non-typical triglycerides, fatty acids and long-chain esters such as 1,2-dioctanoin, 1,3-dicaprin, geranyl oleate, neryl palmitate, oleic acid, linoleic acid, and isopropyl palmitate. These constituents are characteristic of fixed oils or cosmetic emollients, rather than steam-volatile essential oils, and therefore support the conclusion that some products are diluted or extended with lipidic carriers.

**Table 8.** The top 20 compounds found in the market sample MAR019, which includes a monoacylglycerol ester, diacylglycerols, and other atypical esters that are not present in authenticated turmeric rhizome essential oils.

Market Sample MAR019	
Compound	%
ar-Turmerone	18.37
α-Turmerone	18.36
Unidentified	12.33
β-Turmerone	9.75
1-Hydroxy-3-(octanoyloxy)propan-2-yl decanoate	9.15
1,2-Dioctanoin	6.21
β-Sesquiphellandrene	4.18
α-Zingiberene	3.98
ar-Curcumene	3.27
1,3-Dicaprin	2.72
trans-α-Atlantone	1.82
2,4,4,6-Tetramethyl-6-phenyl-1-heptene	1.12
β-Bisabolene	0.93
Cyclohexanecarboxylic acid 3-phenylpropyl ester	0.85
6S,7R-Bisabolone	0.83
Ethyl-2-hexanoate triglyceride	0.74
trans-γ-Atlantone	0.69
1,8-Cineole	0.49
β-Caryophyllene	0.46
Turmeronol A	0.40

These compositional irregularities reflect compromised product quality and the variability present in the predominantly steady market. Overall, while the majority of turmeric rhizome essential oils analyzed appeared to contain the typical major constituents of *C. longa* oil, the occurrence of atypical products indicative of dilution with fatty esters, blending with other essential oils, or the presence of substantial unidentified fractions, highlights the need for compositional verification and quality control within the essential oil supply chain. Indeed, 4/25 of the market samples were confirmed as inauthentic turmeric essential oil (MAR008, MAR013, MAR017, MAR019). Typical constituent presence and abundance are major indicators of essential oil quality, and greatly impact the safety and efficacy of essential oils (Salgueiro et al. 2010).

### Turmeric essential oil safety review

Turmeric essential oil is generally recognized as safe (GRAS) with an oral LD₅。 in rats over 5 g/kg, indicating that the substance is practically nontoxic under the Hodge and Sterner scale *via* oral exposure (Hodge and Sterner 1949; Liju et al. 2013). Similarly, dermal toxicity studies in rabbits revealed an LD₅。 greater than 5 g/kg, supporting the classification of turmeric essential oil as practically nontoxic when applied to the skin (Opdyke and Letizia 1983). Although when applied undiluted under occlusion it is slightly irritating to the ab2013raded skin of rabbits, when applied at 4% to the forearms of 25 volunteers in a maximization study, no irritating or sensitizing effects were observed (Opdyke and Letizia 1983). Additionally, turmeric essential oil was determined not to be phototoxic (Opdyke and Letizia 1983).

Rats administered turmeric essential oil by gavage at doses of 100, 250, and 500 mg/kg body weight per day for 13 weeks showed no adverse effects based on assessments of general condition and behavior, hematological parameters, and histopathological examinations (Liju et al. 2013). The No Observed Adverse Effect Level (NOAEL) was determined to be greater than 500 mg/kg bw/day.

Two independent Ames tests reported negative findings (Hastak et al. 1997; Park 2002; Liju et al. 2013). Three *in vivo* studies, comprising a micronucleus test, a chromosome aberration assay, and a comet assay yielded negative results, suggesting an absence of genotoxic potential (Liju et al. 2013).

The oral use of 0.6 ml turmeric essential oil daily for a month, followed by 1 ml daily for two months, in nine healthy participants was well tolerated. Two participants withdrew, one due to skin rashes and another due to recurrent fever requiring antibiotic treatment. The remaining seven participants reported mild adverse effects, including abdominal discomfort, and headaches. There were no significant changes in heart rate, blood pressure, body weight, hematological tests, liver function tests or kidney function tests. In lipid profile testing, six volunteers showed no change in fasting sugar, cholesterol, triglycerides, HDL or LDL. In one volunteer, triglycerides and LDL were normal at 4 weeks, but elevated at 12 weeks. Levels had returned to normal one month after discontinuing the turmeric oil. There was no change in the menstrual pattern of the three female volunteers (Joshi et al. 2003). Thirty clinical studies focused on formulations designed to enhance the bioavailability of turmeric extract constituents. Three of these specifically evaluated the combined use of turmeric oil and extract, reporting a 6.93- to 8.2-fold increase in curcumin bioavailability compared to turmeric extract alone (Antony et al. 2008; Sunagawa et al. 2015; Mukkadan 2021). Reported doses of turmeric essential oil ranged from 7.5 to 600 mg/day, although seventeen studies did not disclose the exact amount of turmeric essential oil used. Study durations varied from single-dose administration to continuous dosing over 24 months. In ten studies, no adverse events (AEs) were reported; seven studies did not mention AEs; and the remaining studies described mild AEs such as skin rashes, restlessness, tingling sensations, headaches, dizziness, sleepiness, anorexia, pruritus, constipation, flatulence, and mild heartburn (Hastak et al. 1997; Chandran and Goel 2012; Lopresti et al. 2014; Sanmukhani et al. 2014; Lopresti et al. 2015; Hejazi et al. 2016; Sterzi et al. 2016; Amalraj et al. 2017; Gopi et al. 2017; Mikirova et al. 2017; Haroyan et al. 2018; Purpura et al. 2018; Adibian et al. 2019; Hodaei et al. 2019; Saadati et al. 2019; Shep et al. 2019; Arun et al. 2020; Purbadi et al. 2020; Raj et al. 2020; Karandish et al. 2021; Lopresti et al. 2021; Petracca et al. 2021; Singhal et al. 2021; Al-Askar et al. 2022; Santosa et al. 2022; Soni et al. 2022; Thavorn et al. 2024). In all cases, these AEs were not significantly different between the treatment and control groups.

One study evaluated the topical application of a cream containing turmeric essential oil (the amount of turmeric essential oil was not specified) in patients with head and neck cancer to reduce radiation-induced dermatitis over a seven-week period. AEs were not reported (Palatty et al. 2014). Two clinical trials evaluated the inhalation of turmeric essential oil in healthy individuals and patients with COVID-19. In one study, 50 µL of the oil was placed near the participants’ noses once; while the other study lasted two months, but the dosage was not specified. AEs were either not reported or unknown (Sattayakhom et al. 2021; Karpishchenko et al. 2023). Finally, one study investigated the use of turmeric essential oil microballoons as a perfusion/embolization treatment for primary liver cancer. The exposure amount and duration of treatment were not specified, though it was described as short-term. Reported AEs included reductions in white blood cell count, platelet count, and hemoglobin levels, as well as nausea and vomiting. These effects were less severe in the treatment group compared to controls and were considered tolerable by the patients (Kai Xu et al. 2005).

The available data supports a favorable safety profile for turmeric (*C. longa*) essential oil as practically nontoxic in acute oral and dermal LD50 studies. It is also not phototoxic and not genotoxic. Limited evidence suggests the oil is nonirritating nor sensitizing. While the subchronic oral NOAEL above 500 mg/kg/day in rats suggests an acceptable daily intake (ADI) above 500 mg/person/day for an adult, the oil has been used at doses up to 1 g/person/day in a clinical setting with mild adverse effects. Altogether, although some of the toxicological endpoints have not been formally evaluated, the data overall supports the safety of turmeric essential oil.

### Turmeric essential oil pharmacology review

Turmeric essential oil has historically been overshadowed by the more extensively studied curcuminoids. However, growing evidence highlights the oil as a bioactive natural complex substance with potent anti-inflammatory, antimicrobial, neuroprotective, and antioxidant activities, primarily attributed to its sesquiterpenes including ar-turmerone, α-turmerone, and β-turmerone (curlone) (Funk et al. 2010; Stanojević et al. 2015; Amalraj et al. 2017).

Anti-inflammatory effects of turmeric essential oil are well documented *in vitro* and *in vivo. In vitro*, ar-turmerone reduces inflammation through suppression of TNF-α and inflammatory cytokines, inhibition of NF-κB, JNK, and p38 MAPK signaling pathways, and activation of NRF-2 and heme oxygenase-1 in microglial cells (Park et al. 2012). Ar-turmerone also inhibits inflammatory cytokine production in lymphocyte cultures (Oh et al. 2014). *In vivo*, ar-turmerone ameliorates psoriasis-like inflammation in mice by decreasing epidermal hyperplasia and immune infiltration (Li et al. 2018), and turmeric-derived compounds show preventive potential against inflammation-related carcinogenesis (Onuma et al. 2011). When pretreated with turmeric essential oil, mice with induced inflammation showed reduction of oxidative stress markers and enhanced recovery following lipopolysaccharide-induced inflammation (Rana et al. 2015). Clinical data on turmeric essential oil, though limited, parallel these results. Patients with osteoarthritis or rheumatoid arthritis showed significant pain reduction and improved joint function following supplementation with bioavailable curcuminoid and turmeric oil formulations (Amalraj et al. 2017; Singhal et al. 2021). These outcomes support turmeric essential oil’s role as a complementary anti-inflammatory agent.

The antimicrobial potential of turmeric essential oil spans bacteria, fungi, and viruses. Its volatile constituents inhibit *Staphylococcus aureus*, *Escherichia coli*, *Candida albicans*, *Aspergillus flavus* and other pathogens (Negi et al. 1999; Dhingra et al. 2007; Singh et al. 2011; Ferreira et al. 2013; Jankasem et al. 2013; Afifi and Elshenawy 2022; Li et al. 2023). An *in vitro* study by Yu et al. (2025) suggests that mechanisms of action include increased membrane permeability and suppression of key enzymes involved in microbial energy metabolism and redox homeostasis. Turmeric essential oil may also demonstrate antiviral activity, showing that sesquiterpene constituents including ar-turmerone have high binding affinity for the COVID-19 virus (Mahomoodally et al. 2021). Notably, turmeric oil and turmerone-rich extracts demonstrate activity against multidrug-resistant strains, both alone and in combination with antibiotic therapy (Afifi and Elshenawy 2022; Yuandani et al. 2024).

Turmeric essential oil also exhibits neurotrophic and neuroprotective effects, primarily through ar-turmerone. In cell and animal models, turmerones stimulate neural stem cell differentiation and proliferation, promote neurite outgrowth, prevent deprivation-induced apoptosis, inhibit acetylcholinesterase activity, and protect against glutamate- or amyloid-β–induced toxicity (Fujiwara et al. 2010; Park et al. 2012; Hucklenbroich et al. 2014; Saga et al. 2020). These effects suggest possible improved neuroprotection of dopaminergic neurons in neurodegenerative disease models (Hori et al. 2021). An *in vivo* study in an Alzheimer’s disease mouse model showed that ar-turmerone improved learning and memory performance, corresponding with reduced neuroinflammation and improved levels of acetylcholine (Oh et al. 2025). Little clinical evidence of turmeric essential oil’s neuroprotective or neurotropic effects exists in clinical research, although initial studies using formulations of curcumin combined with turmeric oil suggest that this may be a rich avenue of exploration (Rainey-Smith et al. 2016).

Research indicates that antioxidant mechanisms of turmeric essential oil operate both directly and indirectly. Turmerone-rich oils neutralize reactive oxygen species and enhance endogenous defense systems by elevating SOD, catalase, and glutathione peroxidase while reducing pro-inflammatory cytokines and chemokines, PGE2, iNOS, and reactive oxygen species (Park et al. 2012; Ezzatkhah et al. 2023). In animal models, these activities conferred antinociceptive effects in induced acute and chronic inflammation states (Liju et al. 2011). Multiple antioxidant pathways influenced by turmeric essential oil resulted in reduced damage following ischemia and reperfusion in a rat model (Dohare et al. 2008). Studies in inflammatory gut models suggest that ar-turmerone downregulates colonic inflammatory cytokines, influences intestinal flora, and helps protect against induced damage (Li et al. 2022). This effect is also seen in preparations including both curcumin and turmeric essential oil (Toden et al. 2017). Human studies corroborate reductions in oxidative stress biomarkers and improved health outcomes following supplementation with curcumin enhanced with turmeric essential oil (Amalraj et al. 2017). Two human trials on cancer patients with radiation-induced oral mucositis have demonstrated that bioavailable turmeric formulations including both curcuminoids and turmeric essential oil can reduce pain, dysphagia, inflammation, and weight loss significantly better than placebo (Arun et al. 2020; Soni et al. 2022).

Anti-cancer activity of turmeric essential oil and its key volatile components, particularly ar-turmerone, is noted through multiple mechanisms. Compounds from *C. longa* have demonstrated anti-proliferative, apoptotic, and immunomodulatory effects *in vitro* (Yue et al. 2010). Ar-turmerone selectively induces apoptosis in leukemia and lymphoma cells (Aratanechemuge et al. 2002; Lee 2009), suppresses breast cancer cell invasion by downregulating MMP-9 and COX-2 *via* NF-κB inhibition (Park et al. 2012), and demonstrates anti-angiogenic properties *in vitro* and *in vivo* (Yue et al. 2015). In glioma cells, ar-tumerone inhibited proliferation and mobility through suppression of cathepsin B and inhibition of P27 cleavage (Cao et al. 2023). It also enhances immune activation and antitumor responses *in vivo* (Kim et al. 2013). Turmerones and other non-curcuminoid constituents of turmeric show complementary bioactivity to curcumin and hold promise for cancer therapy and drug delivery formulations (Nair et al. 2019). Recent reviews reaffirm turmeric’s broad anticancer potential, emphasizing synergistic effects among its diverse bioactive compounds (Kunnumakkara et al. 2023). Clinical studies on turmeric essential oil and its constituents are scarce, but early safety studies point to its suitability for phase II or III anti-cancer human studies (Joshi et al. 2003). In an *ex vivo* study involving cells cultured from patients with oral submucous fibrosis, turmeric essential oil demonstrated chemoprotective effects against benzo[a]pyrene induced DNA damage (Hastak et al. 1997). A small prospective case series found that oral turmeric oil following antimicrobials promoted lasting regression of low-grade squamous intra-epithelial lesions and prevented progression to higher-grade lesions over 36 months (Joshi et al. 2016). Further research is needed in this area.

Beyond its core pharmacological domains, turmeric essential oil and its main constituents demonstrate gastroprotective, hepatoprotective, cardioprotective, anti-convulsant, anti-depressant, and wound-healing properties (Prakash et al. 2011; Liao et al. 2013; Orellana-Paucar et al. 2013; Syafri et al. 2024; Higashihara et al. 2025). Animal studies show improved lipid profiles in both rat and Syrian hamster models (Ling et al. 2012; Singh et al. 2013). Additional evidence points to antidiabetic, anti-obesity, and antivenom actions (Ferreira et al. 1992; Takemoto et al. 2022) indicating broad potential therapeutic scope. One clinical study also found positive results in reducing facial hair growth in females using a 1% turmeric oil formulation (Majeed et al. 2025). Another study investigated aromatic turmeric essential oil, measuring patient EEG data during inhalation, and found that the aroma was associated with more positive mood (Sattayakhom et al. 2021). Turmeric’s extensive therapeutic potential continues to be actively investigated, lending scientific support to its centuries-long use in traditional medicine systems.

In addition to its own bioactivity, turmeric essential oil may also serve as a significant bioavailability enhancer of curcumin, enhancing absorption in human intestinal epithelial cells (Yue et al. 2012). Multiple clinical studies with formulations of standardized curcumin extracts and other botanicals combined with volatile turmeric essential oil suggest that the essential oil enhances bioavailability which may translate into improved therapeutic outcomes (Antony et al. 2008; Hodaei et al. 2019; Arun et al. 2020; Soni et al. 2022).

Despite strong experimental evidence, clinical validation remains limited by small sample sizes and variability in oil composition. Standardized formulations with defined turmerone content, coupled with pharmacokinetic characterization, will be critical to establishing reproducibility and regulatory approval (Li et al. 2011). Nonetheless, the convergence of mechanistic and translational data supports turmeric essential oil as a distinct and complementary bioactive fraction of turmeric. Its favorable safety profile and growing clinical validation suggest utility in managing inflammation-driven, infectious, and neurodegenerative conditions. Continued refinement of formulations and high-quality human clinical studies will be pivotal in unlocking the full therapeutic opportunities of turmeric’s volatile constituents.

## Conclusions

Turmeric (*C. longa*) rhizome essential oil represents a well-studied natural complex substance. Its quality is influenced by agricultural conditions, post-harvest processing, and distillation methodology. Comprehensive analytical evaluation of the essential oil confirms that turmerones are by far the major constituents in authentic rhizome oil, while monoterpene abundance is generally reduced in oil produced from ground turmeric compared to oil from fresh turmeric. A market survey revealed substantial variability among commercial products. Although some can be attributed to natural variability, some products were clearly diluted with non-essential oil material. Toxicological evidence demonstrates a favorable safety profile, with low acute toxicity, absence of dermal toxicity or genotoxicity, and good tolerability in both animals and humans. Pharmacological studies highlight diverse bioactivity properties, including anti-inflammatory, antimicrobial, neuroprotective, antioxidant, and anticancer effects, largely attributable to turmerone-rich fractions. Despite promising therapeutic potential, clinical evidence for turmeric essential oil remains limited. Altogether, the current body of evidence supports turmeric essential oil as a safe and multifunctional natural product with significant potential for therapeutic and nutraceutical applications. Continued high-quality clinical studies will be critical to fully define the essential oil’s clinical relevance. Finally, what this study has highlighted is the need for well characterized and authenticated essential oils to ensure safety and efficacy as well as the reproducibility in future research.

## Supplementary Material

Turmeric essential oil_Supplementary Information_DRAFT03.docx

## Data Availability

All data supporting the findings of this study are included within the main text of the article and its supplementary files.
