# Supplementary material for: Turmeric (curcuma longa) rhizome essential oil: analytical profile of authenticated and commercial samples, safety and pharmacology review
Source: Pharm Biol. 2026 Mar 1;64(1):379–97. doi: 10.1080/13880209.2026.2629622 (PMC12954815; doi:10.1080/13880209.2026.2629622)
Supplement: Turmeric essential oil_Supplementary Information_DRAFT03.docx [file IPHB_A_2629622_SM6892.docx]

**Table S1**: Main constituents (%, including constituents present at ≥0.5% in at least one sample, and unique compounds) of essential oils of turmeric from 25 commercial market samples.

| **Compound** | **MAR001** | **MAR002** | **MAR003** | **MAR004** | **MAR005** | **MAR006** | **MAR007** | **MAR008** | **MAR009** | **MAR010** | **MAR011** | **MAR012** | **MAR013** | **MAR014** | **MAR015** | **MAR016** | **MAR017** | **MAR018** | **MAR019** | **MAR020** | **MAR021** | **MAR022** | **MAR023** | **MAR024** | **MAR025** |
| --- | --- | --- | --- | --- | --- | --- | --- | --- | --- | --- | --- | --- | --- | --- | --- | --- | --- | --- | --- | --- | --- | --- | --- | --- | --- |
| ar-Turmerone | 23.18 | 28.35 | 24.33 | 21.66 | 28.89 | 25.07 | 23.28 | 0.18 | 29.37 | 23.65 | 20.90 | 25.38 | 21.44 | 33.37 | 33.62 | 25.68 | 4.34 | 32.17 | 18.37 | 27.63 | 32.05 | 29.42 | 26.55 | 17.36 | 17.85 |
| α-Turmerone | 14.08 | 18.28 | 13.75 | 13.13 | 18.25 | 14.50 | 9.99 | 0.12 | 14.66 | 12.88 | 9.95 | 20.06 | 23.68 | 15.25 | 17.15 | 23.78 | 8.05 | 22.14 | 18.36 | 13.37 | 9.55 | 12.30 | 13.51 | 15.55 | 33.78 |
| β-Turmerone | 5.91 | 17.44 | 6.03 | 6.40 | 12.85 | 12.59 | 4.15 | 0.30 | 15.16 | 12.18 | 4.07 | 13.41 | 13.05 | 14.53 | 16.26 | 8.82 | 4.10 | 14.59 | 9.75 | 12.04 | 4.08 | 5.94 | 12.77 | 7.55 | 15.80 |
| Unidentified | 13.97 | 2.77 | 14.15 | 7.34 | 7.60 | 1.01 | 16.69 | 30.24 | 1.96 | 2.22 | 20.36 | 12.94 | 5.15 | 1.50 | 2.10 | 12.28 | 2.03 | 6.36 | 12.33 | 2.88 | 8.41 | 12.84 | 0.18 | 6.72 | 0.85 |
| α-Zingiberene | 6.70 | 4.57 | 5.87 | 8.78 | 4.10 | 6.66 | 5.23 |  | 4.66 | 6.14 | 5.35 | 5.06 | 6.01 | 4.45 | 4.01 | 6.40 | 1.68 | 3.84 | 3.98 | 5.50 | 6.04 | 6.32 | 6.11 | 10.63 | 7.02 |
| β-Sesquiphellandrene | 3.22 | 6.41 | 3.03 | 6.17 | 5.82 | 8.53 | 2.43 | 0.24 | 7.47 | 8.61 | 2.47 | 3.73 | 6.08 | 7.32 | 6.42 | 3.71 | 1.75 | 3.20 | 4.18 | 7.70 | 4.80 | 4.66 | 8.77 | 8.30 | 7.10 |
| ar-Curcumene | 6.05 | 3.84 | 5.01 | 8.06 | 6.35 | 5.50 | 5.52 |  | 5.24 | 5.35 | 5.47 | 2.97 | 4.19 | 5.67 | 4.34 | 3.07 | 0.71 | 3.06 | 3.27 | 5.59 | 8.77 | 5.91 | 5.61 | 7.54 | 2.84 |
| Octan-2-yl palmitate |  |  |  |  |  |  |  |  |  |  |  |  |  |  |  |  | 73.50 |  |  |  |  |  |  |  |  |
| trans-α-Atlantone | 2.02 | 3.65 | 2.03 | 1.61 | 2.84 | 2.49 | 1.60 |  | 3.62 | 2.22 | 1.50 | 3.23 | 2.94 | 3.14 | 2.84 | 2.57 | 0.62 | 2.89 | 1.82 | 2.57 | 1.59 | 1.60 | 1.92 | 1.13 | 2.46 |
| β-Bisabolene | 1.98 | 1.19 | 1.59 | 2.57 | 1.53 | 2.14 | 1.88 |  | 1.55 | 1.92 | 1.93 | 1.02 | 1.43 | 1.40 | 1.13 | 1.20 | 0.30 | 0.81 | 0.93 | 1.82 | 2.06 | 1.70 | 1.58 | 2.17 | 1.22 |
| 1,8-Cineole | 0.97 | 0.46 | 1.31 | 1.97 | 0.48 | 1.62 | 1.40 | 0.36 | 0.80 | 3.71 | 1.56 | 0.57 | 0.92 | 0.69 | 0.57 | 0.48 | 0.28 | 0.45 | 0.49 | 3.44 | 1.93 | 0.99 | 3.38 | 2.17 | 1.01 |
| γ-Curcumene | 3.85 | 0.15 | 2.97 | 1.78 | 0.34 | 0.16 | 4.39 |  | 0.14 | 0.13 | 4.45 | 0.93 | 0.47 | 0.10 | 0.08 | 1.31 | 0.10 | 0.57 | 0.29 |  | 1.62 | 1.82 | 0.11 | 1.57 | 0.38 |
| α-Phellandrene | 0.96 | 0.83 | 1.50 | 2.87 | 0.10 | 0.57 | 1.61 |  | 0.89 | 1.66 | 1.86 | 0.63 | 0.38 | 0.73 | 0.85 | 0.35 | 0.21 | 0.48 | 0.18 | 0.73 | 1.40 | 1.00 | 1.37 | 2.37 | 0.76 |
| 2,4,4,6-Tetramethyl-6-phenyl-1-heptene | 2.65 | 0.81 |  | 1.88 | 1.51 | 0.33 |  |  | 0.73 | 0.34 |  | 0.77 | 1.68 | 0.67 | 0.57 | 0.62 | 0.15 | 0.77 | 1.12 | 0.42 |  | 3.27 | 0.35 | 1.78 | 0.63 |
| 6S,7R-Bisabolone | 0.62 | 1.69 | 0.69 | 0.49 | 1.53 | 1.68 | 0.71 |  | 1.98 | 1.56 | 0.63 | 0.95 | 1.41 | 1.61 | 1.37 | 0.67 | 0.27 | 0.80 | 0.83 |  | 0.30 | 0.53 | 1.27 | 0.34 | 1.08 |
| para-Cymene | 0.96 | 0.53 | 1.32 | 2.05 | 0.17 | 0.55 | 1.48 | 0.07 | 0.77 | 1.15 | 1.58 | 0.54 | 0.32 | 0.71 | 0.81 | 0.19 | 0.09 | 0.52 | 0.17 | 0.91 | 1.85 | 0.92 | 0.99 | 2.01 | 0.31 |
| Terpinolene | 0.51 | 0.06 | 0.62 | 1.21 | 0.19 | 0.04 | 0.56 | 0.07 | 0.07 | 0.03 | 0.58 | 0.36 | 0.43 | 0.05 | 0.04 | 0.29 | 0.25 | 0.27 | 0.23 |  | 1.04 | 0.84 | 0.02 | 2.26 | 0.90 |
| β-Caryophyllene | 1.33 | 0.61 | 1.14 | 1.78 | 0.70 |  | 1.16 |  | 0.61 |  | 1.28 | 0.47 | 0.71 | 0.43 | 0.51 | 1.00 | 0.18 | 0.47 | 0.46 |  | 1.34 | 1.00 |  | 2.64 | 0.73 |
| trans-γ-Atlantone | 2.02 |  | 2.15 | 0.66 | 0.44 |  | 2.30 |  | 0.13 |  | 2.00 | 1.24 | 0.92 |  |  |  | 0.07 | 1.10 | 0.69 |  | 1.08 | 0.74 |  | 0.25 | 0.26 |
| Helifolen-12-al B |  |  | 2.68 |  |  |  | 4.62 |  |  |  | 4.35 |  |  |  |  |  |  |  |  |  | 3.43 |  |  |  |  |
| Geranial |  |  |  |  |  |  |  | 14.51 |  | 0.02 |  |  |  |  |  |  |  |  |  |  |  |  |  |  |  |
| Biotol |  | 1.44 |  |  | 0.33 | 1.32 |  |  | 1.40 | 1.19 |  |  | 0.20 | 1.33 | 1.15 |  | 0.14 |  |  | 1.04 |  |  | 1.09 |  | 0.34 |
| ar-Turmerol |  | 0.92 |  |  | 0.52 | 1.59 |  |  | 1.22 | 0.94 |  |  | 0.34 | 1.07 | 0.93 |  | 0.05 |  | 0.25 | 1.88 |  |  | 1.80 |  | 0.23 |
| Limonene | 0.15 | 0.09 | 0.33 | 0.37 | 0.51 | 0.13 | 0.26 | 4.19 | 0.10 | 1.23 | 0.27 | 0.09 | 0.08 | 0.08 | 0.10 | 0.05 | 0.03 | 0.08 | 0.04 | 0.95 | 0.26 | 0.13 | 0.98 | 0.28 | 0.10 |
| cis-α-Atlantone | 0.56 | 0.81 | 0.72 | 0.38 | 0.54 | 0.60 | 0.40 |  | 0.79 |  | 0.51 | 1.09 | 0.55 | 0.24 | 0.35 | 0.78 | 0.05 | 0.20 |  | 0.63 |  |  |  | 0.31 | 0.41 |
| cis-γ-Atlantone | 0.84 | 0.06 | 0.85 | 0.66 | 0.47 | 0.09 | 0.94 |  | 0.21 |  | 0.85 | 0.71 | 0.55 | 0.13 |  | 0.97 | 0.04 | 0.61 | 0.31 |  | 0.49 | 0.66 |  | 0.23 | 0.21 |
| trans-γ-Bisabolene | 0.64 | 0.41 | 0.56 | 0.74 | 0.31 | 0.68 | 0.58 |  | 0.40 | 0.53 | 0.49 | 0.32 | 0.32 | 0.20 | 0.19 | 0.27 | 0.08 | 0.17 | 0.18 | 0.39 | 0.40 | 0.33 | 0.40 | 0.42 | 0.40 |
| 1-Hydroxy-3-(octanoyloxy)propan-2-yl decanoate |  |  |  |  |  |  |  |  |  |  |  |  |  |  |  |  | 0.10 |  | 9.15 |  |  |  |  |  |  |
| Isobicyclogermacrene |  |  |  |  |  | 2.27 |  |  | 0.18 | 1.69 |  |  |  |  | 0.22 |  |  |  |  | 1.41 |  |  | 1.47 |  |  |
| α-Santalene |  |  |  | 0.53 |  | 1.92 |  |  |  | 1.47 |  |  |  | 0.10 | 0.19 |  |  |  |  | 1.28 | 0.58 | 0.54 | 1.33 | 0.35 |  |
| Amorpha-4,11-diene | 0.94 | 0.07 | 0.74 | 0.80 | 0.20 | 0.06 | 0.99 |  | 0.07 | 0.04 | 1.02 | 0.34 | 0.18 |  |  | 0.40 | 0.03 | 0.26 | 0.12 |  | 0.64 | 0.64 |  | 0.82 | 0.13 |
| Geranyl oleic acid |  |  |  |  |  |  |  | 7.65 |  |  |  |  |  |  |  |  |  |  |  |  |  |  |  |  |  |
| Neral |  |  |  |  |  |  |  | 7.56 |  |  |  |  |  |  |  |  |  |  |  |  |  |  |  |  |  |
| 6R,7R-Bisabolone | 0.42 | 0.04 | 0.48 | 0.23 | 0.22 | 0.07 | 0.49 |  | 0.08 |  | 0.42 | 0.47 | 0.24 |  |  | 0.51 |  | 0.37 | 0.13 | 1.37 | 0.30 | 0.26 |  |  | 0.08 |
| α-Humulene | 0.28 | 0.38 | 0.24 | 0.53 | 0.24 | 0.30 | 0.26 |  | 0.26 | 0.25 | 0.26 | 0.19 | 0.30 | 0.21 | 0.31 | 0.28 | 0.08 |  | 0.20 | 0.15 | 0.32 | 0.44 | 0.17 | 0.80 | 0.31 |
| β-Curcumene | 0.73 | 0.10 | 0.51 | 0.63 | 0.17 | 0.09 | 0.56 |  | 0.12 | 0.13 | 0.66 | 0.28 | 0.26 | 0.09 | 0.07 | 0.32 | 0.05 | 0.20 | 0.14 |  | 0.37 | 0.39 |  | 0.47 | 0.31 |
| Cucumadione | 0.39 | 0.22 | 0.34 | 0.40 | 0.45 | 0.31 | 0.34 |  | 0.24 | 0.25 | 0.33 | 0.25 | 0.22 | 0.22 | 0.29 | 0.23 | 0.03 | 0.21 | 0.16 | 0.55 | 0.30 | 0.37 | 0.40 | 0.23 | 0.21 |
| trans-β-Farnesene |  | 0.35 |  | 0.24 | 0.21 | 0.80 |  |  | 0.48 | 0.67 |  | 0.10 | 0.23 | 0.39 | 0.33 | 0.11 | 0.09 |  | 0.14 | 0.50 | 0.24 | 0.19 | 0.57 | 0.48 | 0.33 |
| 1,2-Dioctanoin |  |  |  |  |  |  |  |  |  |  |  |  |  |  |  |  |  |  | 6.21 |  |  |  |  |  |  |
| Zingiberenol |  | 0.56 |  |  |  | 0.84 |  |  | 0.72 | 0.77 |  |  |  | 0.62 | 0.58 |  | 0.03 |  |  | 0.67 |  |  | 0.70 |  | 0.06 |
| 6-Methyl-5-hepten-2-one |  |  |  |  |  |  |  | 0.91 |  | 0.03 |  |  |  |  |  |  |  |  |  |  |  | 0.01 | 0.02 | 0.01 |  |
| Germacrene B |  |  |  |  |  | 0.56 |  |  |  | 0.64 |  |  |  |  | 0.29 |  |  |  |  | 0.42 |  |  | 0.54 |  |  |
| Linoleic acid |  |  |  |  |  |  |  | 3.93 |  |  |  |  | 1.62 |  |  |  | 0.06 |  |  |  |  |  |  |  |  |
| Oleic Acid |  |  |  |  |  |  |  | 4.38 |  |  |  |  | 1.13 |  |  |  |  |  |  |  |  |  |  |  |  |
| Citronellal |  |  |  |  |  |  |  | 4.64 |  |  |  |  |  |  |  |  |  |  |  |  |  |  |  |  |  |
| Citronellyl acetate |  |  |  |  |  |  |  | 3.54 |  |  |  |  |  |  |  |  |  |  |  |  |  |  |  |  |  |
| 1,3-Dicaprin |  |  |  |  |  |  |  |  |  |  |  |  |  |  |  |  |  |  | 2.72 |  |  |  |  |  |  |
| Neryl palmitic acid |  |  |  |  |  |  |  | 2.15 |  |  |  |  |  |  |  |  |  |  |  |  |  |  |  |  |  |
| Citronellic acid |  |  |  |  |  |  |  | 1.75 |  |  |  |  |  |  |  |  |  |  |  |  |  |  |  |  |  |
| Citronellyl palmitoleic acid |  |  |  |  |  |  |  | 1.33 |  |  |  |  |  |  |  |  |  |  |  |  |  |  |  |  |  |
| Palmitic acid |  |  |  |  |  |  |  | 0.52 |  |  |  |  | 0.52 |  |  |  |  |  |  |  |  |  |  |  |  |
| Isopropyl myristate |  |  |  |  |  |  |  | 0.92 |  |  |  |  |  |  |  |  |  |  |  |  |  |  |  |  |  |
| Geranyl acetate |  |  |  |  |  |  |  | 1.06 |  |  |  |  |  |  |  |  |  |  |  |  |  |  |  |  |  |
| Geranyl stearic acid |  |  |  |  |  |  |  | 0.81 |  |  |  |  |  |  |  |  |  |  |  |  |  |  |  |  |  |
| Geranyl Linolenic acid |  |  |  |  |  |  |  | 0.77 |  |  |  |  |  |  |  |  |  |  |  |  |  |  |  |  |  |
| Isopropyl palmitate |  |  |  |  |  |  |  |  |  |  |  |  |  |  |  |  | 0.10 |  |  |  |  |  |  |  |  |
| Ethyl-2-hexanoate triglyceride |  |  |  |  |  |  |  |  |  |  |  |  |  |  |  |  |  |  | 0.74 |  |  |  |  |  |  |

**Figure S1.** Representative chromatograms of (a) ground and (b) fresh turmeric rhizome essential oil and adulterated samples (c) MAR008, (d) MAR017, (e) MAR013, (f) MAR019.

1. **Turmeric Ground**

1. **Turmeric Fresh**

1. **MAR008**

1. **MAR017**

1. **MAR013**

1. **MAR019**
